# The Treatment of Neuralgia of the Trigeminal Nerve

**Published:** 1893-06

**Authors:** 


### The Treatment of Neuralgia of the Trigeminal Nerve.

It is not necessary to define neuralgia, but a trigeminal neuralgia has certain peculiarities. The anatomy of the Gasserian ganglion and its three branches is known to all, but the difficulty of getting at them in the living person is little appreciated except by those who have tried. This neuralgia differs from ordinary neuralgia in being paroxysmal and is probably the most painful that one has to do with. There are periods of absolute quiet between the paroxysms. The changes seen in the parts supplied by this nerve and its branches are very slight in proportion to the pain the patient feels. There is generally no change at all or at times a slight glazed appearance of the skin and very rarely there has been atrophy of the muscles supplied by this nerve.

The age at which this form of neuralgia occurs is a most important etiological factor. It generally occurs in persons over forty, but one case in a woman of twenty-seven has been reported. Another etiological factor is, that there are certain points of pain where the nerve makes its exit through the bone. When the surgeon has been called in, the treatment by drugs has been exhausted. Quinine and arsenic are drugs that do the most good and it was formerly supposed for this reason that these neuralgias were malarial, but the more recent discovery of the plasmodium malariæ has shown that this is not always so. The next best drug is aconitia pushed to its full physiological effect; also antipyrin and antefebrin have been used with good effect. The surgical methods are section of the nerve, stretching, and excision more or less far back. These are the three surgical methods used at the present day. Excision of the nerve was first done by Carnochan in 1858, and his operation was classical. The patient on whom he operated was a physician of the State of Maryland, in Caroline County. His operation was successful. Obalinski operated on a case several times but relief was not permanent. There are numerous ways of getting at the nerve. We may take it from in front or from behind. Rose and Anderson of Chicago, were the first to take it from behind. Horsley has also done it in this way.

Tiffany has operated twice in the head for intractable neuralgia. Both cases had been operated on before and one several times. In one case excision of the upper jaw nerve had been done. In his first case, which was a woman, he went through the side of the head, exposed the side of the head, raised up the dura mater until he arrived at the middle division of the fifth, put a string around it so as not to lose it, then scraped back the dura with a dental instrument which he had slightly dulled, and brought the ganglion into sight. It was a novel sight to him to see the Gasserian ganglion exposed to view in a living person. The lower division of the fifth nerve sends a motor branch to the muscles of mastication and in operating it is very hard to leave this motor branch untouched. When the head is first opened the brain swells up and fills the entire space and it seems almost impossible to get at the nerve, but when the shock came on in this case the brain shrunk and got smaller and made operating possible. He did not pour cerebro-spinal fluid or blood into the head as had been recommended, and he did not suture the bone but the fascia. He wounded the dura but no harm came of it. The patient got well with no temperature. There was at first entire anæsthesia of the parts supplied by the middle and upper branches of the nerve, but there was no paralysis at all and pain ceased from the time she went under the anæsthetic and she had no suffering at all. For two days the dressings were soaked with cerebro-spinal fluid from the wound but he took off the dressings and took out the wire on the third day and everything was healed. The patient has been kept under observation for some time after the operation, and she has had no relapses. *Am. Med. Ass. Jour.*

